# Proximal and distal movement patterns during a graphomotor task in typically developing children and children with handwriting problems

**DOI:** 10.1186/s12984-021-00970-9

**Published:** 2021-12-20

**Authors:** Shoshana Steinhart, Patrice L. Weiss, Jason Friedman

**Affiliations:** 1 ALYN Pediatric and Adolescent Rehabilitation Center, Shmaryahu Levin Street, Kiryat Yovel, Jerusalem, Israel; 2 Department of Occupational Therapy, Faculty of Social Welfare and Health Sciences, University of Haifa, Haifa, Israel; 3 Department of Physical Therapy, Stanley Steyer School of Health Professions, Sackler Faculty of Medicine, Tel Aviv University, Tel Aviv, Israel; 4 Sagol School of Neuroscience, Tel Aviv University, Tel Aviv, Israel

**Keywords:** Handwriting, Movement analysis, Proximal, Distal joints, Stability, Motor control

## Abstract

**Background:**

Therapists specializing in handwriting difficulties in children often address motor problems including both proximal and distal movements in the upper extremity. Kinematic measures can be used to investigate various aspects of handwriting. This study examined differences in movement patterns in proximal and distal joints of the upper extremity during graphomotor tasks between typically developing children with and without handwriting problems. Additionally, it explored relationships between movement patterns, speed, and legibility of writing.

**Methods:**

Forty-one children, aged 7–11 years, were assessed with the Aleph Aleph Ktav Yad Hebrew Handwriting assessment and the Beery Test of Visual Motor Integration and, based on their scores, were divided into a research group (with handwriting difficulties) and a control group (without handwriting difficulties). Upper extremity joint movement patterns were analyzed with a motion capture system. Differences in the quality of shapes traced and copied on a graphics tablet positioned horizontally and vertically were compared. Between-group differences and relationships with speed and legibility were analyzed.

**Results:**

In both groups, there was greater movement in the distal compared to the proximal joints, greater movement when performing the task in a horizontal compared to a vertical plane, and greater movement when tracing than copying. Joint movements in the arm executed scaled-down versions of the shapes being drawn. While the amount of joint displacement was similar between groups, children in the research group showed greater dissimilarity between the drawn shape and the shape produced by the proximal joints. Finally, the drawing measure on the tablet was a significant predictor of legibility, speed of writing, visual motor integration and motor coordination, whereas the dissimilarity measure of joint movement was a significant predictor of speed of writing and motor coordination.

**Conclusions:**

This study provides support for the role of the distal upper extremity joints in the writing process and some guidance to assist clinicians in devising treatment strategies for movement-related handwriting problems. While we observed differences in proximal joint movements between the children with and without handwriting difficulties, the extent to which they are responsible for the differences in drawing quality remains to be determined. Further studies should use a similar methodology to examine additional tasks such as drawing shapes of varying sizes.

## Background

Handwriting and drawing are major occupations for children, both in the classroom and at home. Elementary school children often spend between 30 and 60% of their school day involved in fine motor activities, 85% of which involve handwriting and drawing tasks [1–3]. Despite the increased use of computers and other types of communication technology, handwriting [4] continues to be a main occupation for children in the classroom [2]. Indeed, the time spent on paper and pencil tasks increases substantially from kindergarten to elementary school [4]. Writing legibly and efficiently is essential for later academic achievement, allowing children to express, communicate, and record ideas, and may have an effect on a child’s self-esteem [5–7]. Writing is a very complex skill, involving both lower-level perceptual-motor processes and higher-level cognitive process skills. A handwriting problem can therefore result from multiple factors including difficulties in language, cognitive, visual perceptual, visual motor integration and fine motor skills [8–10].

Handwriting legibility and handwriting kinetics have been shown to be related to in-hand manipulation skills [11–13]. Moreover, a stable base of support in the proximal upper extremity, i.e., the shoulder and elbow, appears necessary for distal control of the wrist, thumb and fingers [14, 15] to position the handwriting tool properly in space and provide support and control over its motion [16].

Up to 27% of children in elementary school are reported to have handwriting problems [10, 17, 18], making it one of the most common reasons for referral of a school-aged child to occupational therapy [5, 19]. Occupational therapists primarily focus on motor skills including strengthening of proximal (shoulder girdle) and distal (intrinsic hand) muscles. Therapists treating children for handwriting difficulties often work under the assumption that proximal control is a prerequisite for manipulative hand use [15, 20].

Kinematic and kinetic measures have been used to investigate various aspects of the writing process in a more objective manner than traditional handwriting assessments, allowing an analysis of the process of writing as opposed to focusing on the product [5, 21–26]. Children with both handwriting and other motor difficulties use faster stroke velocities to write sequences of simple characters [21]. Furthermore, less proficient writers display higher movement velocities and a higher incidence of undesirable movements, thus demonstrating a greater expenditure of energy [26]. Although children with motor coordination and handwriting problems have difficulty performing movements required for handwriting [21], no studies were found that examined specific upper extremity movement patterns during handwriting and whether these movements provide stability or control.

Miyahara et al. [27], using an electromagnetic tracking system to monitor 3D kinematics, found that inaccurate drawers demonstrate more coincidental proximal movements in the head and shoulders when making drawing errors; inaccurate drawers seem to be unable to adjust their posture for drawing, perhaps due to their difficulty in coordinating activity in the head, shoulder and arm. This suggests that proximal stability is important for fine motor control.

Data from an EMG-digitizer system showed that different strategies are used when performing tracing and copying tasks in different planes of motion; there is greater activation of proximal muscles when the task is performed in a vertical versus the transverse (horizontal) plane. Nevertheless, task accuracy was similar in the two planes suggesting that the product can look similar despite a change in movement strategy [28].

On the other hand, increased variability in both kinematic components and muscle activity may indicate an excess of movement which is unnecessary to achieve a legible final product. Greater utilization of energy would therefore be required and this may bring about a less optimal product, resulting in either poorer quality or slower writing, as found in other studies [29].

It is important for health professionals working with children on handwriting to understand the underlying motor components influencing the handwriting process, and specifically to be able to identify which proximal and distal upper extremity movement patterns lead to greater efficiency in writing. Few studies have examined this topic with the notable exception of Lacquaniti et al. [30]'s examination of the relationships between proximal and distal amplitudes of movements and shape size during drawing tasks in adults. They found that the relative roles of proximal and distal joints vary with the size of the shape being drawn. While occupational therapists often address both proximal and distal components influencing handwriting, a review of the literature did not reveal any studies that examined differences in proximal and distal movement patterns during graphomotor tasks between children with and without handwriting impairments.

The aims of this study were to: (1) Identify differences in movement patterns of the proximal and distal upper extremity in typically developing children and children with handwriting difficulties during two graphomotor tasks (copying versus tracing) and on two different surface orientations (vertical versus horizontal surfaces) and (2) Examine the relationship between features of proximal and distal upper extremity movements and writing legibility and speed in children with and without handwriting disabilities. Understanding the contributions of the different upper extremity joints can help therapists determine which joints are used to control the drawing of a shape, and which joints provide stability during a graphomotor task. Moreover, defining differences between children with and without handwriting problems can direct clinicians in deciding which impairments to focus on in treatment.

## Methods

### Participants

Forty-one children, aged 7–10 years (2nd to 5th grade), participated in the study. The participants were a convenience sample from a summer camp for typically developing children during the summers of 2018 and 2019 and from a list of children waiting to receive occupational therapy for handwriting difficulties at the ALYN Hospital Pediatric and Adolescent Rehabilitation Center. Children were excluded from the study if they did not have sufficient cognitive ability to copy a paragraph, had other diagnoses such as intellectual and developmental disability (IDD), were on the autism spectrum, had any reported neurological or perceptual-motor problems, or were receiving physical or occupational therapy for developmental disabilities other than handwriting.

Participants were divided into two groups: research and control, based on their handwriting proficiency as determined by the Aleph Aleph Ktav Yad (AAKY) Hebrew Handwriting test [31]; children in the research group scored above the AAKY cutoff scores in two areas. The two groups were matched for age and sex. There were 20 participants in the control group (8 males, 12 females, mean age = 8.5 years) and 21 in the research group (11 males, 10 females, mean age = 8.8 years). The children in the research group had significantly lower scores on the Beery motor coordination subtest than children in the control group as well as on three out of four parts of the AAKY (See Table 1 for details).

**Table 1:** Demographic information

Quantity	Control group (N = 20)	Research group (N = 21)	p-value (t-test / chi squared)
Male/Female	8/12	11/10	0.751^#^
Mean age (years)	8.5 (range 8–10.8)	8.8 (range 7.1–10.8)	0.146
Hand dominance (right/left)	17/3	18/2	1.00^#^
Beery VMI	29 (15)	19 (15)	0.039
Beery motor	46 (23)	15 (13)	<0.001*
Letters per minute (copying)	40.5 (14.0)	24.7 (8.6)	<0.001*
Illegible letters (copying)	5.0 (2.9)	9.1 (5.0)	0.003*
Letters per minute (dictation)	54.9 (16.0)	38.6 (9.7)	<0.001*
Illegible letters (dictation)	7.4 (3.2)	10.8 (4.9)	0.014

Values in brackets are standard deviations unless otherwise stated. # = category data, Pearson’s χ^2^ test, * = significantly different (p < 0.05). The p-values were corrected for multiple comparisons using the Holm method

Instruments:

1. The Ascension trakSTAR System was used to measure movements in the different upper extremity joints, including proximally in the trunk, shoulder, and elbow, and distally in the wrist, thumb and fingers. This is a magnetic motion capture system that records the 3D location and orientation of miniature cylindrical sensors (2.0 mm outer diameter × 9.9 mm length) placed above and below each joint to be measured. The joint angles of the arm (wrist, elbow and shoulder) were calculated offline.
2. Intuos Pro Wacom Graphics Tablet (43 cm x 28 cm) was used to measure kinematic properties of movement during graphomotor tasks with a wireless inking stylus pen and A4 paper affixed to the tablet in which the children could see their writing on the paper itself.

Tests

1. Aleph-Aleph Ktav Yad (AAKY) Hebrew handwriting test [31] was used to measure speed and quality of handwriting. The AAKY is a reliable and valid Hebrew script handwriting test used extensively in Israel by occupational therapists [31, 32]. Speed of handwriting is measured by counting the number of letters written in one minute and the amount of time taken to write a 106-letter paragraph. Legibility is measured by determining the number of unidentifiable letters (defined as letters that the examiner is unable to identify at first glance when differentiated from the rest of the word) that make it more difficult to read the paragraph accurately and by looking at spatial organization on the paper which includes spacing, sizing, attention to margins and alignment on writing guidelines. Each subitem of spatial organization is scored by the examiner on a 4-point scale (1 being best and 4 being worst) based on specific criteria and are then added together for a total score for spatial organization. Higher scores in spatial organization correspond to poorer performance. Children are identified as having a handwriting problem when they score above the cutoff scores for their grade level in two areas (including number of illegible letters, time taken to write a paragraph, letters per minute, organization on paper, and number of corrections).
2. Beery- Butenika Developmental Test of Visual Motor Integration (VMI) (5th edition) [33] is a paper-and-pencil test that presents a developmental sequence of geometric forms that are copied. It was used to test the child’s visual motor integration (VMI subtest) and motor coordination skills (motor coordination subtest). It has norms from age 2 through 99 years [33]. In the visual motor integration subtest, the child copies a series of shapes beginning from basic shapes, gradually getting more complex. Each shape is scored by the examiner as correct or incorrect based on specific criteria. In the motor coordination subtest, the child draws the shapes within a path of the same shape, and is scored correct if they do not go out of the lines. A total raw score is recorded for each subsection and translated to a standard score and percentile based on the child’s age.

### Procedure

**Fig. 1:**
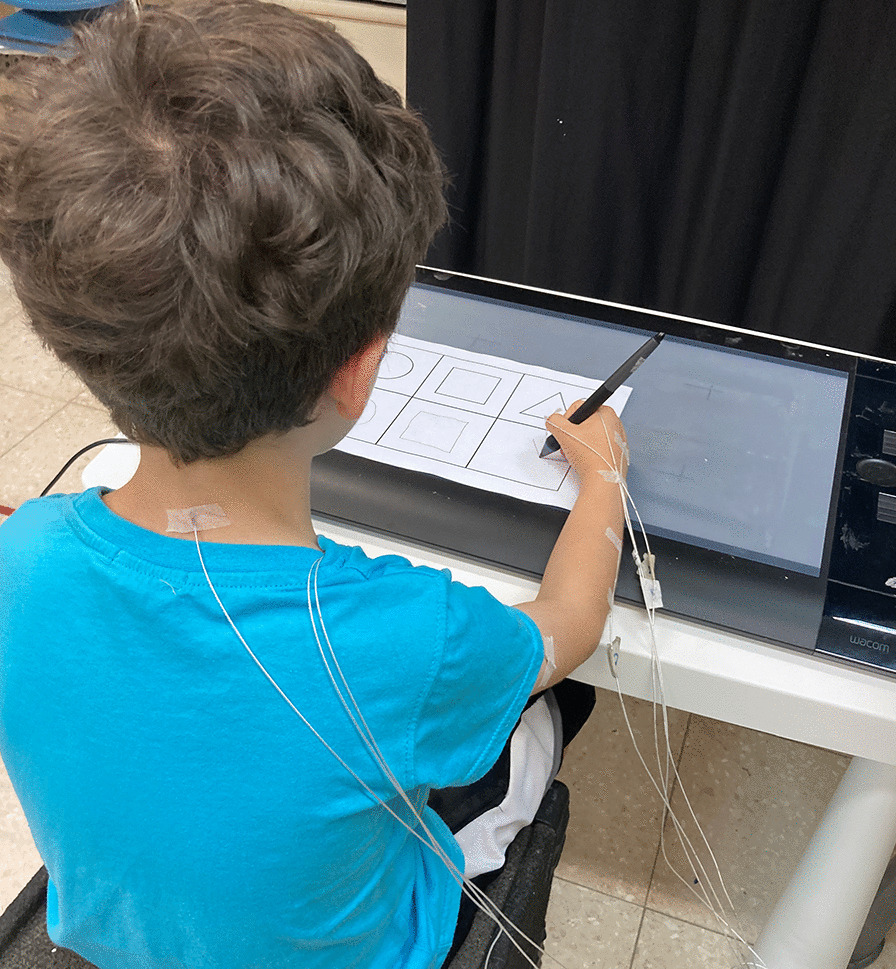
The experimental setup. The participant performed the task on a Wacom graphics tablet, while Ascension trakStar sensors were taped to 7 locations (two of the sensors are under the clothing). Note that the tablet used in this illustrative photo is different from the tablet used in the experiment

Following obtainment of parental informed consent and child assent, the child was seated at a height-adjustable desk and chair with feet resting on the floor, hips and knees at 90 degrees, with elbows resting at 90 degrees on the desk surface (see Fig. 1). An experienced occupational therapist administered the AAKY Hebrew handwriting test, the VMI and motor coordination components of the Beery test. Following a brief rest period, seven trakSTAR sensors were placed on the dominant hand at the following locations: Distal joints: thumbnail, index fingernail, dorsal surface of hand halfway between the metacarpal phalangeal and wrist joints, dorsal surface of forearm halfway between the wrist and elbow, Proximal joints: dorsal surface of upper arm halfway between shoulder and elbow, on the acromion, and between the scapulae in the middle of the upper back at the level of the vertebrae T1-T2 (see Fig. 1). The Wacom tablet was placed 3 cm from the edge of the table nearest to the child. The children first practiced by writing their names with a wireless pen stylus on a piece of paper affixed to the tablet. They were then asked to copy two sentences from the AAKY, to copy a circle, square and triangle from a displayed photo, and to trace a circle, square, and triangle. These tasks were performed in random order four times with and without the trakSTAR sensors while the tablet was placed horizontally on the table surface and while it was placed vertically on an incline board.

### Data and statistical analysis

We calculated four outcome measures based on the data recorded from the tablet—two measures that quantified the quality of the drawing, and two measures that quantified the joint displacement of the arm. To quantify the drawing quality, we calculated the scale (i.e., how large the drawing was compared to the displayed shape) and the drawing error (the extent to which the drawn shape differed from the actual shape). This best-fit of the drawn shape (circle, square or triangle) was determined by finding the optimal changes in location and scale such that the differences between the best-fit and actual shape were minimal, using a technique described previously [28, 34]. We then compared the best-fit shapes to the actual shapes and calculated two drawing variables: relative scale (1 = same size, 0.5 = half size) and drawing error (mean difference between the drawn shape from its best-fit shape, in percent of the height of the best-fit shape). For both variables, we first performed a mixed design ANOVA with the sensors as a factor (i.e., with or without the trakSTAR sensors) and examined the interaction of group and sensors to test whether the sensors affected the relative scale and error measure due, for example, to encumbrance or wire interference, and whether this differed between groups.

**Fig. 2:**
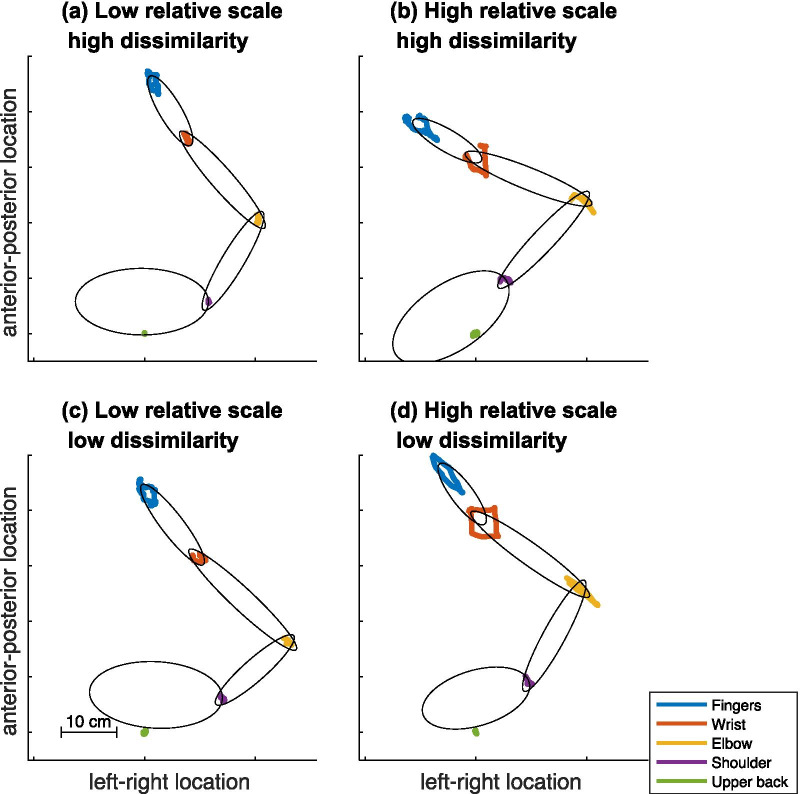
Sample displacements of the fingers, wrist, elbow, shoulder and upper back, as shown from above, for copying a square in the horizontal orientation as described above. Panel (a) includes a sketch of the body (trunk, upper arm, lower arm, hand) to aid in understanding the figure, note that this is not based on actual recordings of the body size rather ellipses fit to the joint locations

With regard to quantifying displacements of the arm joints, we used the trakSTAR data to calculate the magnitude of the displacement of each joint and the dissimilarity of the joint trajectories relative to the drawn shapes. First, the locations of the wrist, elbow and shoulder joints were identified by determining the transformation from the markers on either side of the relevant joint to a single point across time (i.e., the center of rotation) [35]. We also computed the mean location of the thumb and index finger sensors and the sensor on the upper back. For these five locations, we compared the shape drawn on the tablet with the displacement of the relevant location while drawing the shape (see Fig. 2). In particular, we compared the amount of movement in a given plane by calculating the area of an ellipse that included 95% of the data (using principal component analysis to find the ellipse that captured the most data). We then compared the area of this ellipse to that of the tablet data (1 = same size, 0.5 = half size) which we refer to as the magnitude variable. Additionally, we compared how similar the shapes were by finding the optimal transformation of the scaled shape (i.e., after adjusting their size so that their ellipse areas were the same), using a Matlab function (pcregistericp) for point cloud registration and calculating the root mean square error between the closest points in the shapes, which we refer to as the dissimilarity variable. The Matlab codes used in the analyses as well as all the data collected are available online [36].

We analyzed the four variables (scale, drawing error, magnitude, and dissimilarity) using four mixed-design ANOVAs, with between-group factor: group (children with and without handwriting difficulties), and within-group factors: shape (circle, square, triangle), orientation (horizontal, vertical) and task (copying, drawing). For magnitude and dissimilarity, there was also a within-group factor of joint (finger, wrist, elbow, shoulder, upper back). In addition, we used a backward stepwise linear regression with a removal criterion of p = 0.1 [37] to test whether the four variables were able to predict drawing ability as defined by letters per minute and number of illegible letters in copying and dictation, and the Beery visual motor integration and motor coordination scores.

## Results

### Tablet data

The mixed design ANOVA did not reveal any significant differences between performance (scale or drawing errors) with (mean ± SD: scale: 0.882 ± 0.069; drawing error: 2.380 ± 0.735) or without (scale: 0.899 ± 0.068; drawing error: 2.458 ± 0.653) the trakSTAR sensors (scale: F(1,29)= 3.901, p = 0.058; drawing error: F(1,29)= 1.501, p = 0.230), nor was there a significant interaction with group observed (scale: F(1,29)=0.006, p = 0.938; drawing error: F(1,29)=0.314, p = 0.579). The data across these trials were therefore collapsed for the remainder of the analyses.

The results of the mixed-design ANOVA are reported in Tables 2 and 3. For the scale measure, only a main effect of task was found (F(1,29) = 61.927, p < 0.001); when tracing, the size of the drawn shapes (0.984 ± 0.013) was significantly closer to the size of the original shape compared to copying (0.793 ± 0.130, p < 0.001).

**Table 2:** Mean ± Standard deviation for the scale and drawing error measures, averaged by orientation, task, shape and group

Group	Horizontal						Vertical					
	Copy			Trace			Copy			Trace		
	$\circ$	$\square$	$\triangle$	$\circ$	$\square$	$\triangle$	$\circ$	$\square$	$\triangle$	$\circ$	$\square$	$\triangle$
Scale												
Control	0.808 ± 0.166	0.807 ± 0.166	0.798 ± 0.147	0.991 ± 0.012	0.992 ± 0.008	0.976 ± 0.012	0.810 ± 0.154	0.796 ± 0.164	0.778 ± 0.139	0.988 ± 0.013	0.992 ± 0.005	0.975 ± 0.008
Research	0.780 ± 0.160	0.813 ± 0.120	0.795 ± 0.156	0.981 ± 0.011	0.988 ± 0.019	0.967 ± 0.053	0.776 ± 0.130	0.814 ± 0.161	0.800 ± 0.154	0.982 ± 0.012	0.991 ± 0.011	0.972 ± 0.024
Drawing error (%)												
Control	2.835 ± 1.478	3.450 ± 1.511	3.278 ± 1.483	0.801 ± 0.295	0.974 ± 0.504	0.790 ± 0.192	3.347 ± 1.355	4.471 ± 2.427	3.448 ± 1.503	0.780 ± 0.273	0.897 ± 0.357	0.836 ± 0.282
Research	3.718 ± 1.283	4.147 ± 1.422	3.847 ± 1.282	1.264 ± 0.833	1.238 ± 0.562	1.281 ± 0.621	3.861 ± 1.496	5.016 ± 2.448	4.245 ± 1.955	1.306 ± 0.712	1.258 ± 0.682	1.063 ± 0.385

**Table 3:** Results of mixed-design ANOVA on scale, drawing error and movement time. Three way interactions (all non-significant) are not shown

Factor	F	p	Comparison (when significant)
*Scale*			
Orientation	F(1,29) = 0.250	0.621	
Task	F(1,29) = 61.927	<0.001	Copy (0.793 ± 0.130) < Trace (0.984 ± 0.013)
Shape	F(2,58) = 2.943	0.061	
Group	F(1,29) = 0.167	0.686	
Orientation * Group	F(1,29) = 0.910	0.348	
Task * Group	F(1,29) = 0.476	0.496	
Shape * Group	F(2,58) = 0.482	0.620	
Orientation * Task	F(1,29) = 0.246	0.624	
Orientation * Shape	F(2,58) = 0.173	0.842	
Task * Shape	F(1,29) = 0.996	0.375	
*Drawing error*			
Orientation	F(1,29) = 1.930	0.175	
Task	F(1,29) = 605.5	<0.001	Trace (1.045 ± 0.477) < Copy (3.903 ± 0.912)
Shape	F(2,58) = 5.483	0.007	[Circle (2.269 ± 1.006), Triangle (2.340 ± 0.875) ]< Square (2.546 ± 0.960)
Group	F(1,29) = 7.422	0.011	Control (2.149 ± 0.436) < Research (2.694 ± 0.544)
Orientation * Group	F(1,29) = 0.570	0.456	
Task * Group	F(1,29) = 0.403	0.530	
Shape * Group	F(2,58) = 0.590	0.558	
Orientation * Task	F(1,29) = 2.620	0.116	
Orientation * Shape	F(1,29) = 1.556	0.220	
Task * Shape	F(1,29) = 4.348	0.017	Trace: no difference [Circle (1.056 ± 0.602), Square (1.091 ± 0.517), Triangle (1.018 ± 0.415)] Copy: [Circle (3.531 ± 1.370), Triangle (3.735 ± 1.292)] < Square (4.325 ± 1.426)

For the drawing error measure, several main effects and an interaction were found. A main effect of task was observed (F(1,29) = 605.5, p < 0.001); overall, the children made much larger errors (nearly 4 times greater) when copying (3.903 ± 0.912) compared to tracing (1.045 ± 0.477, p < 0.001). A main effect of shape was also observed (F(2,58) = 5.483, p = 0.007); the square (2.546 ± 0.960 was drawn significantly less accurately than either the circle (2.269 ± 1.006, p = 0.017) or the triangle (2.340 ± 0.875, p = 0.017). Finally, a main effect of group was observed (F(1,29) = 7.422, p = 0.011); the magnitude of the errors was lower for children in the control group (2.149 ± 0.436) compared to the research group (2.694 ± 0.544). Additionally, an interaction between task and shape was found (F(1,29) = 4.348); all shapes showed similar drawing errors for the tracing task (Circle (1.056 ± 0.602), Square (1.091 ± 0.517), Triangle (1.018 ± 0.415), p > 0.05 for all comparisons), whereas for the copying task, the square had larger drawing errors (4.325 ± 1.426) than the Circle (3.531 ± 1.370, p < 0.001) or the Triangle (3.735 ± 1.292, p = 0.002).

### Joint displacement data

Examples of the magnitude and dissimilarity results are shown in Fig. 2. Panel a shows a sample where the mean magnitude is low (0.08) but with a high mean dissimilarity (30.9 mm). Panel b shows a sample where both mean magnitude (0.42) and mean dissimilarity (24.7 mm) are high. Panel c shows a sample where both mean magnitude (0.28) and mean dissimilarity (9.2 mm) are low. Finally, panel d shows a sample of high magnitude (0.75), but low dissimilarity (6.42 mm).

The summary data are shown in Tables 4 and 5. A main effect for magnitude was observed for orientation (F(1,30) = 6.175, p = 0.019); the joint displacement was significantly greater when performing the task on a horizontal surface (0.499 ± 0.197) than on a vertical surface (0.410 ± 0.111, p = 0.019). A main effect was also observed for joint (F(4,120) = 97.020, p < 0.001); the more distal the joint, the greater the displacement, with the exception of the shoulder and upper back which were not significantly different (Fingers (0.996 ± 0.370) > Wrist (0.596 ± 0.230) >Elbow (0.396 ± 0.200) > Shoulder (0.170 ± 0.119, p < 0.001) and Upper back (0.121 ± 0.097, p < 0.001). A main effect was also observed for task (F(1,30) = 5.846); during tracing, the joint displacement was significantly greater (0.492 ± 0.168) than during copying (0.416 ± 0.183; p =0.022). In addition, there was an interaction between joint and task (F(4,120) = 3.192, p = 0.016); task differences were only observed for the distal joints (fingers and wrist), see Table 4.

**Table 4:** Mean ± Standard deviation for the magnitude and dissimilarity, between drawing on the tablet and the joint movements.

Group	Copy					Trace				
	Upper back	Shoulder	Elbow	Wrist	Fingers	Upper back	Shoulder	Elbow	Wrist	Fingers
Magnitude-horizontal										
Control	0.158 ± 0.163	0.226 ± 0.213	0.354 ± 0.273	0.516 ± 2.289	1.014 ± 0.606	0.146 ± 0.133	0.210 ± 0.147	0.473 ± 0.263	0.711 ± 0.287	1.148 ± 0.296
Research	0.097 ± 0.071	0.154 ± 0.118	0.321 ± 0.207	0.544 ± 0.431	1.035 ± 1.413	0.175 ± 0.224	0.210 ± 0.265	0.416 ± 0.291	0.733 ± 0.513	0.987 ± 0.321
Magnitude-vertical										
Control	0.133 ± 0.193	0.164 ± 0.140	0.358 ± 0.287	0.432 ± 0.173	0.806 ± 0.472	0.099 ± 0.079	0.144 ± 0.094	0.445 ± 0.304	0.704 ± 0.319	0.945 ± 0.313
Research	0.070 ± 0.070	0.085 ± 0.064	0.306 ± 0.157	0.421 ± 0.177	0.668 ± 0.183	0.068 ± 0.056	0.100 ± 0.075	0.369 ± 0.253	0.734 ± 0.359	1.049 ± 0.322
Dissimilarity (mm)–horizontal										
Control	22.1 ± 11.0	19.9 ± 7.7	15.2 ± 7.7	9.7 ± 3.7	7.4 ± 1.7	23.0 ± 11.7	16.8 ± 5.7	14.0 ± 4.8	9.2 ± 3.8	6.5 ± 2.2
Research	30.2 ± 26.7	31.0 ± 16.5	16.3 ± 7.2	11.0 ± 4.0	8.6 ± 1.8	24.4 ± 13.0	30.4 ± 15.4	18.0 ± 9.1	12.6 ± 7.8	7.6 ± 3.3
Dissimilarity (mm)–vertical										
Control	27.2 ± 16.7	22.3 ± 8.7	14.2 ± 3.9	11.8 ± 4.4	7.9 ± 2.0	27.4 ± 141	26.5 ± 13.0	16.1 ± 8.5	11.0 ± 5.4	6.5 ± 2.1
Research	41.3 ± 24.8	36.1 ± 23.1	15.0 ± 5.0	12.8 ± 7.7	9.0 ± 4.2	43.7 ± 47.4	30.2 ± 18.7	16.5 ± 10.9	10.2 ± 6.4	6.9 ± 2.6

The values are averaged over the three shapes

For the dissimilarity measure, a main effect of joint was also observed (F(4,120) = 64.772, p < 0.001) with the more proximal joints showing larger dissimilarity; shoulder (25.7 ± 10.4) and upper back (29.4 ± 14.8) were greater than elbow (15.3 ± 4.7), which was greater than fingers (7.5 ± 1.6) and wrist (11.0 ± 3.5). A main effect was observed for group (F(1,30) = 8.091, p = 0.008) with the research group showing significantly more dissimilarity (33.9 ± 11.2) than the control group (26.4 ± 4.7). However, an interaction of group and joint (F(4,120) = 4.632, p = 0.002) showed that the difference between the groups was only significant for the proximal joints (shoulder: control (21.4 ± 5.6) < research (31.5 ± 11.7 and upper back: control (25.2 ± 9.6) < research (34.5 ± 18.1)). In addition, an interaction of group and task (F(1,30) = 5.218, p = 0.03) showed that a significant difference between the groups was observed only for the copying task (control (16.0 ± 3.6) < research (21.3 ± 7.9)]) and not for the tracing task (control (15.7 ± 3.6), research (19.2 ± 7.1)]). Other main effects and interactions (Shape, Orientation * Joint, Task * Shape) are reported in Table 4.

### Regression analysis

We tested how well the kinematic measures were able to predict the outcomes of functional tests, specifically the number of letters per minute, number of illegible letters, as well as the Beery VMI, and the Beery Motor Coordination subtests. The results of the regression analysis are reported in Table 6. The drawing error was a significant negative predictor for all four measures. Dissimilarity was a significant negative predictor for letters per minute and the Beery motor score only. In addition, age was a significant positive predictor for letters per minute and number of illegible letters, and female participants had significantly higher Beery motor scores than males.

**Table 5:** Results of mixed-design ANOVA on magnitude and dissimilarity, in comparing the drawing on the tablet with the joint movements

Factor	F	p	Comparison (when significant)
Magnitude			
Orientation	F(1,30) = 6.175	0.019	Vertical (0.410 ± 0.111) < Horizontal (0.499 ± 0.197)
Joint	F(4,120) = 97.020	<0.001	Fingers (0.996 ± 0.370) > Wrist (0.596 ± 0.230) >Elbow (0.396 ± 0.200) > [Shoulder (0.170 ± 0.119), Upper back (0.121 ± 0.097)]
Task	F(1,30) = 5.846	0.022	Copying (0.416 ± 0.183) < Tracing (0.492 ± 0.168)
Shape	F(2,60) = 1.561	0.218	
Group	F(1,30) = 0.009	0.927	
Orientation * Group	F(1,30) = 0.097	0.757	
Joint * Group	F(4,120) = 0.622	0.648	
Task * Group	F(1,30) = 0.684	0.415	
Shape * Group	F(2,60) = 0.421	0.658	
Orientation * Joint	F(4,120) = 2.107	0.084	
Orientation * Task	F(1,30) = 0.211	0.650	
Orientation * Shape	F(2,60) = 1.674	0.196	
Joint * Task	F(4,120) = 3.192	0.016	Only Fingers [Copying (0.946 ± 0.635) < Tracing (1.039 ± 0.281)] and Wrist [Copying (0.487 ± 0.203) < Tracing (0.700 ± 0.340)] different. Others not different: Elbow [Copying (0.357 ± 0.225), Tracing (0.428 ± 0.241)], Shoulder [Copying (0.169 ± 0.136±), Tracing (0.171 ± 0.134±)] Upper back [Copying (0.122 ± 0.113), Tracing (0.122 ± 0.110)]
Joint * Shape	F(8,240) = 1.955	0.053	
Task * Shape	F(1,30) = 2.741	0.073	
*Dissimilarity*			
Orientation	F(1,30) = 2.990	0.094	
Joint	F(4,120) = 64.772	<0.001	[Fingers (7.5 ± 1.6), Wrist (11.0 ± 3.5)] <Elbow (15.3 ± 4.7) < [Shoulder (25.7 ± 10.4), Upper back (29.4 ± 14.8)]
Task	F(1,30) = 2.734	0.109	
Shape	F(2,60) = 6.686	0.002	Circle (19.1 ± 6.5) > [Square (16.9 ± 7.8), Triangle (17.2 ± 6.1)]
Group	F(1,30) = 8.091	0.008	Control (26.4 ± 4.7) < Research (33.9 ± 11.2)
Orientation * Group	F(1,30) = 0.033	0.858	
Joint * Group	F(4,120) = 4.632	0.002	No difference for Fingers [Control (7.1 ± 1.3), Research (7.9 ± 1.8)], Wrist [Control (10.5 ± 3.0), Research (11.6 ± 4.0)], Elbow [Control (14.9 ± 4.5), Research (16.2 ± 4.8)]. Shoulder [Control (21.4 ± 5.6) < Research (31.5 ± 11.7)] Upper back [Control (25.2 ± 9.6) < Research (34.5 ± 18.1)]
Task * Group	F(1,30) = 5.218	0.030	Trace: no difference [Control (15.7 ± 3.6), Research (19.2 ± 7.1)] Copy: [control (16.0 ± 3.6) < Research (21.3 ± 7.9)]
Shape * Group	F(2,60) = 0.186	0.831	
Orientation * Joint	F(4,120) = 3.444	0.011	Upper back: Horizontal (24.4 ± 14.6) < Vertical (34.3 ± 21.0) Other joints: no difference: shoulder: [Horizontal (23.6 ± 12.3), Vertical (28.1 ± 14.4)] elbow: [Horizontal (15.4 ± 5.9), Vertical (15.2 ± 5.2)] wrist: [Horizontal (10.6 ± 4.2), Vertical (11.3 ± 4.4)] fingers: [Horizontal (7.6 ± 2.0), Vertical (7.5 ± 2.2)]
Orientation * Task	F(1,30) = 0.010	0.920	
Orientation * Shape	F(2,60) = 0.845	0.435	
Joint * Task	F(4,120) = 0.884	0.476	
Joint * Shape	F(8,240) = 0.818	0.517	
Task * Shape	F(1,30) = 11.581	<0.001	Triangle: copy (21.0 ± 10.9) < trace (13.6 ± 3.5) Other shapes: no difference: circle: [copy (17.2 ± 8.2), trace (20.8 ± 8.0)] square: [copy (16.8 ± 5.9), trace (17.3 ± 11.6)]

Three-way interactions (all non-significant except for joint * shape * task for both tasks, and orientation * joint * task for relative ellipse area) and four-way interactions (all non-significant) are not shown

**Table 6:** Results of multiple linear regression analysis for predicting letters per minute, illegible letters, Beery visual motor score, and Beery motor score

Predictor	B	p
*Letters per minute copying*		
Age	8.702	<0.001
Drawing error	− 7.402	0.016
Dissimilarity	− 0.471	0.015
*Letters per minute dictation*		
Age	7.129	0.003
Drawing error	− 12.108	<0.001
*Illegible letters copying*		
Drawing error	3.630	0.006
*Illegible letters dictation*		
Age	2.199	0.016
Drawing error	2.251	0.094
*Beery visual motor*		
Drawing error	− 13.528	0.001
*Beery motor*		
Drawing error	− 22.452	<0.001
Dissimilarity	− 0.561	0.091
Sex (0 = male, 1 = female)	10.109	0.097

All regressions are significant (p<0.05), only predictors remaining at the end of the backward stepwise process (i.e., that have p < 0.1) are included.

## Discussion

This study is the first, to our knowledge, to examine associations between quantitative measures of drawing quality (shape accuracy and errors) and upper arm kinematics during graphomotor tasks made by typically developing children and children with handwriting problems. The results revealed greater errors in copying tasks than in tracing tasks, and children with handwriting problems demonstrated less accuracy drawing shapes than typically developing children. For both groups, movement was larger in the distal joints than in the proximal joints, when tasks were performed on a horizontal plane than on a vertical plane and when tracing than when copying. Furthermore, children with handwriting problems demonstrated greater dissimilarity between shapes made distally than with the proximal joints compared to typically developing children. Finally, the drawing variables recorded on the tablet were significant predictors of legibility, speed of writing, visual motor integration and motor coordination; in contrast, the dissimilarity measure of joint movement was a significant predictor of speed of writing and motor coordination.

The current findings show that children with handwriting difficulties make less accurate shapes as assessed by both standardized paper-and-pencil tests and by more objective tablet data than typically developing children. These differences in drawing were evident not only in the shapes drawn on the tablet, but also in the scaled versions made by displacements of the upper arm joints. These findings suggest a relationship between proximal joint movements and handwriting quality; identifying the mechanisms behind this relationship requires further research.

As expected, the distal segments of the arm moved more than the proximal parts, as observed in other studies [29]. This suggests that there may be greater stability in the proximal joints and greater mobility in the distal joints during graphomotor tasks, as reduced displacement is generally associated with greater stability [38]. Furthermore, as the joints became more proximal, the dissimilarity between joint movement and drawn or copied shapes increased, perhaps demonstrating that “responsibility” for achieving the shape is due to decreasing contributions of movement of the fingers, then the wrist, then the elbow and least by the shoulder and trunk. On the other hand, it may be a matter of strategic choice by some children; those who are unable to accurately produce movements in their fingers may utilize compensations in their more proximal joints to produce the shape. This possibility requires further investigation, for example, by experimentally obliging children to use a specific strategy such as constraining shoulder movement, and examining which strategy is most efficient for writing. In keeping with this suggestion, performing a manual task seated as opposed to standing, for example, may provide a child with greater postural stability while completing a graphomotor task thereby reducing their tendency to use trunk compensations [38]. Reducing the degrees of freedom a child needs to perform a task may facilitate stability by limiting the need for greater manual control to achieve accuracy [39].

Miyahara et al. [27] found that inaccurate drawers demonstrate more coincidental proximal movements in their head and shoulders when making drawing errors, and concluded that inaccurate drawing was a result of proximal instability. In contrast, we did not find between-group differences in the magnitude of the shoulder and trunk joint displacements. It should be noted that Miyahara et al. [27] used a different measure for stability (significant extraneous abrupt movement) than the measures used in the current study. We found a difference in the dissimilarity of the joint movements for the shapes at the proximal joints and not in the magnitude of the movements; the children with handwriting difficulties had joint displacements that were more dissimilar to the drawn shape than in the control group. Previous studies of drawing shapes have demonstrated that the shoulder creates a foundation for movement of the entire arm [40]. It appears as though handwriting is also a “whole arm” task such that all of the joints, including the more proximal ones, contribute to the shape and size of the drawn figure. [30]. Sharing the task across multiple arm joints may also improve performance by taking advantage of the redundancy in the degrees of freedom when performing this task [41].

It may be that children who are less proficient writers utilize less control in the proximal joints when drawing a shape; we suggest that the observed movements in this group are more a result of joint reaction forces rather than active control, compared to the control group. It is therefore important for clinicians to consider training the whole arm including the shoulder and utilizing a sensory-motor approach when working on handwriting and other graphomotor tasks. Further studies should explore differences in adopting a more proximal, more distal or uniform strategy during treatment for handwriting.

In addition, there was more joint movement when performing the task on a horizontal surface than on a vertical surface, although there was no difference in accuracy between the two planes. This is similar to findings of other studies which found that although different movement strategies are utilized when drawing on different planes of movement, the accuracy of the product is similar [28].

Another noteworthy finding was that during a tracing task, joint movement is greater than during a copying task, while there were more drawing errors for copying than for tracing. This appears to strengthen the notion that the amount of joint movement does not impact the accuracy of the shape. It appears reasonable that there would be fewer drawing errors during tracing, as tracing is performed on the shape itself and involves primarily motor coordination. In contrast, copying requires higher level cognitive processes such as visual motor integration skills where children need to rely on vision and alternate their gaze between the form that is copied and the form that is produced [42].

It is interesting to note that drawing error was the best predictor of functional outcomes of both speed and legibility, followed by dissimilarity, and that the amount of movement or size of the shape did not predict any of the functional performance outcomes. It appears that children utilize different movement strategies in handwriting, and the amount of movement does not necessarily have an effect on a child’s writing proficiency.

Furthermore, it may be that children with handwriting difficulties have no innate difficulty in controlling joint dynamics, as the difference was found mainly in the copying task and not in the tracing task. Drawing is a complex task involving many skills, including both sensorimotor processes and higher-level cognitive process skills [8–10]. Thus, it may be that the differences that arose in the copying task are primarily related to a higher-level cognitive issue, such as difficulty with planning, imitation or visuomotor transformation, and less dependent on proximal or distal joint dynamics. A limitation of the present study was that movement patterns were examined during tasks that involved copying and tracing basic shapes rather than actual handwriting. This limitation was intentional in an effort to identify associations during such simple, more constrained graphomotor tasks. Further studies are necessary to examine more specific relationships between the different upper extremity joints during handwriting, and whether these relationships differ in children with and without handwriting problems. In addition, our technique of analyzing the shapes drawn by each joint may have been affected by the size of the shapes, as was found previously [30]. Larger shapes may trace 3D paths in space, rather than the largely two-dimensional shapes observed in this study, affecting the outcome variables. We expect that this effect was minor due to the relatively small shapes used; however, to better understand the role of the proximal joints in producing end effector motion, shapes of different sizes should be tested in future studies. This will support an investigation of whether handwriting difficulties are related to recruiting the appropriate arm dynamics for a given task, whether they are due to impairment of proximal muscle control, or whether they are related to higher level cognitive processes. Thus, it is not possible to determine conclusively that proximal joints are responsible for task performance rather than stabilization based on results of this study.

## Conclusions

Understanding motor processes in the upper extremity in greater detail will assist clinicians in devising treatments for handwriting problems related to movement and motor control in the upper extremity joints. The findings of this study appear to provide support for the key contribution of the distal upper extremity joints to the writing process. While we observed differences in proximal joint movements between the children with and without handwriting difficulties, the extent to which they are responsible for the differences in drawing quality remains to be determined. This study enabled identification of various methodological issues that need to be addressed when studying movement patterns during handwriting tasks. Further studies are recommended using a similar methodology to examine additional tasks such as drawing shapes of varying sizes, and adopting different movement strategies in order to reach more definitive conclusions.

## Data Availability

The dataset and code supporting the conclusions of this article are available in the figshare repository, 10.6084/m9.figshare.14922639.
